# Field evidence challenges the often‐presumed relationship between early male maturation and female‐biased sexual size dimorphism

**DOI:** 10.1002/ece3.3450

**Published:** 2017-10-16

**Authors:** Marie‐Claire Chelini, Eileen Hebets

**Affiliations:** ^1^ School of Natural Sciences University of California, Merced Merced CA USA; ^2^ School of Biological Sciences University of Nebraska – Lincoln Lincoln NE USA

**Keywords:** body size, male strategies, mating success, protandry, reproductive timing, sexual size dimorphism, Thomisidae

## Abstract

Female‐biased sexual size dimorphism (SSD) is often considered an epiphenomenon of selection for the increased mating opportunities provided by early male maturation (i.e.*,* protandry). Empirical evidence of the adaptive significance of protandry remains nonetheless fairly scarce. We use field data collected throughout the reproductive season of an SSD crab spider, *Mecaphesa celer*, to test two hypotheses: Protandry provides fitness benefits to males, leading to female‐biased SSD, or protandry is an indirect consequence of selection for small male size/large female size. Using field‐collected data, we modeled the probability of mating success for females and males according to their timing of maturation. We found that males matured earlier than females and the proportion of virgin females decreased abruptly early in the season, but unexpectedly increased afterward. Timing of female maturation was not related to clutch size, but large females tended to have more offspring than small females. Timing of female and male maturation was inversely related to size at adulthood, as early‐maturing individuals were larger than late‐maturing ones, suggesting that both sexes exhibit some plasticity in their developmental trajectories. Such plasticity indicates that protandry could co‐occur with any degree and direction of SSD. Our calculation of the probability of mating success along the season shows multiple male maturation time points with similar predicted mating success. This suggests that males follow multiple strategies with equal success, trading‐off access to virgin females with intensity of male–male competition. Our results challenge classic hypotheses linking protandry and female‐biased SSD, and emphasize the importance of directly testing the often‐assumed relationships between co‐occurring animal traits.

## 1 INTRODUCTION

Females and males often differ in the timing of their reproductive readiness. Such differences in reproductive timing evolve due to differences in the sex‐specific benefits and costs associated with time of maturation, emergence, or arrival on reproductive grounds (see review by Morbey & Ydenberg, 2001; Møller, Balbontín, Cuervo, Hermosell, & De Lope, 2009). In protandrous species—that is, species where males are reproductively ready prior to females—early male maturation is typically related to an increased reproductive success (Aebischer, Perrin, Krieg, Studer, & Meyer, 1996; Morbey, Coppack, & Pulido, 2012). Such an increase in reproductive success is expected to be particularly important in monogamous species or species with first male sperm priority, where early maturation facilitates access to virgin females (Fagerström & Wiklund, 1982; Kvarnemo & Simmons, 2013; Morbey, 2013; Simmons, Llorens, Schinzig, Hosken, & Craig, 1993; Wedell, 1992; Wiklund & Fagerström, 1977; Zonneveld, 1996).

The benefits of early male maturation may be offset by an intense competition for mates, as operational sex ratios early in the reproductive season are strongly male biased (Kasumovic & Andrade, 2009; Parker & Courtney, 1983). The intensity of male–male competition should slowly decrease along the season as more females mature and the sex ratio becomes more equitable, or even female‐biased (Legrand & Morse, 2000; Vollrath & Parker, 1992). In addition, in species where females mate multiply, benefits of protandry will vary depending on the pattern of sperm priority. Synchrony of female maturation also affects the benefits of male protandry. If all females mature in a short window of time, males benefit by maturing earlier. If female maturation is spread along the season, males may find virgin females at any moment, decreasing the benefits of early male maturation (Kasumovic, Bruce, Andrade, & Herberstein, 2008; Parker & Courtney, 1983; Wiklund & Fagerström, 1977).

Across taxa, protandry is often associated with female‐biased sexual size dimorphism (SSD) (Blanckenhorn, 2000; Matsuura, 2006; Morbey & Ydenberg, 2001; Smith & Brockmann, 2014; Vollrath & Parker, 1992). The association between protandry and female‐biased SSD has often been predicted theoretically, (e.g., Abrams, Leimar, Nylin, & Wiklund, 1996; Morbey, 2013; Wiklund & Fagerström, 1977) and observed empirically (e.g., Alcock, 1997; Smith & Brockmann, 2014; Stillwell & Davidowitz, 2010), but what drives their frequent co‐occurrence is still far from clear in many systems (Morbey, 2013). At least two hypotheses have been proposed to explain the joint evolution of protandry and female‐biased SSD (see review by Morbey & Ydenberg, 2001). First, the mating opportunity hypothesis (a form of adaptive protandry) predicts that female‐biased SSD is an indirect by‐product of selection for early male maturation, due to a shortening of male development time (e.g., Alcock, 1997; Candolin & Voigt, 2003). On the other hand, the constraint hypothesis (also called incidental protandry) proposes the opposite, predicting that protandry is a by‐product of selection for another trait, such as large female size or small male size (e.g., Matsuura, 2006). According to the constraint hypothesis, SSD and protandry would only evolve jointly if females and males grow at similar rates, and exhibit little or no plasticity in their growth trajectories. Selection for large female body size (and/or small male body size) would, thus, require longer female growth, with protandry evolving as an indirect consequence (Esperk, Tammaru, Nylin, & Teder, 2007; Tammaru, Esperk, Ivanov, & Teder, 2010; Wiklund, Nylin, & Forsberg, 1991; Zonneveld, 1996). Comparing these two hypotheses, female‐biased SSD may be considered either a cause or a consequence of protandry. Despite the abundance of studies linking these two population traits, there is very little empirical evidence of the adaptive significance of protandry and its potential to drive SSD evolution (Cueva del Castillo & Nunez‐Farfan, 1999; Foellmer & Moya‐Laraño, 2007; Morbey & Ydenberg, 2001). This lack of evidence is in great part due to the difficulties of measuring fitness and growth trajectories in a population‐wide context (Blanckenhorn, 2005).

Spiders are renowned for their frequent female‐biased SSD (Foellmer & Moya‐Laraño, 2007; Head, 1995; Prenter, Elwood, & Montgomery, 1999; Vollrath & Parker, 1992; Wilder, Rypstra, & Elgar, 2009). Many protandrous species of spiders with varying degrees of female‐biased SSD have low remating rates and exhibit no mate choice (Chelini & Hebets, 2016a, 2016b; Johnson, 2005; Maklakov, Bilde, & Lubin, 2004; Morse, 2007; Ramos, Irschick, & Christenson, 2004). Selection for early male maturation and scramble competition for virgin females is therefore often assumed to be the main drivers of SSD (Danielson‐François, Hou, Cole, & Tso, 2012; Dodson & Beck, 1993; Johnson, 2005; Legrand & Morse, 2000; Morse, 2013). In other words, the mating opportunity hypothesis is commonly invoked to explain the evolution of female‐biased SSD in spiders, but this hypothesis is seldom tested empirically. Very little is known about the fitness benefits males derive from protandry, or on the relationship between male size and timing of maturation (Cueva del Castillo & Nunez‐Farfan, 1999; Foellmer & Moya‐Laraño, 2007). In this study, we use detailed field observations compiled along an entire reproductive season combined with a simple and generalizable optimization model to understand the relationship between maturation time, body size, and reproductive success in a female‐biased sexually size dimorphic species of crab spider, *Mecaphesa celer*.

*Mecaphesa celer* is a univoltine flower‐dwelling crab spider. Female *M. celer* are 1.5–2 times the size of males and may weigh up to 10 times the males’ mass (Chelini & Hebets, 2016a, 2016b; Muniappan & Chada, 1970). In the laboratory, female *M. celer* have two to four developmental instars more than males, corresponding to an average difference of 70 days between male maturation and female maturation (Chelini, DeLong and Hebets in prep). Prior studies have found that female *M. celer* are only receptive to remating during a short window of time, with remating rates decreasing from 85% to 15% over 2 days after their first copulation (Chelini & Hebets, 2016a, 2016b). Such results support the mating opportunity hypothesis for the joint evolution of female‐biased SSD and protandry. Nothing is currently known, however, about the degree of protandry, its potential benefits, or on the intensity of male–male competition in the field.

The mating opportunity benefits of protandry depend on the population‐level degree of synchrony in male and female maturation (Kasumovic & Andrade, 2009; Kasumovic et al., 2008). As such, evidence of monogamy and differences in developmental time in the laboratory are not sufficient to support the mating opportunity hypothesis for the joint evolution of female‐biased SSD and protandry. Here, we use field data collected along an entire reproductive season to test both the mating opportunity and the constraint hypothesis in *M. celer* (Table 1). If SSD in *M. celer* is a consequence of selection for adaptive protandry (i.e., mating opportunity hypothesis), then we predict: (1a) males mature synchronously (i.e., in a single peak of low variance) and prior to females in the field; (2a) females mature synchronously and become rapidly mated, so the proportion of virgin females decreases rapidly along the season; (3a) females that mate early in the season have more spiderlings than females mated late in the season and (4a) timing of maturation is directly related to male size, so early‐maturing males should be smaller than late‐maturing males. If, on the other hand, protandry in *M. celer* is a consequence of selection for large female size/small male size (i.e., constraint hypothesis), then we predict: (1b) males tend to mature prior to females, but not in a synchronous fashion, as selection acts on size rather than timing of maturation; (2b) the proportion of virgin females in unrelated to the timing along the season; (3b) large females have more spiderlings than small females, regardless of when along the season do they mate and (4b) as with the mating opportunity hypothesis, timing of maturation is positively related to male size, so early‐maturing males should be smaller than late‐maturing males. Using parameters based on the data we collected while testing the above predictions, we modeled mathematically the probability that an individual female or male would mate according to their timing of maturation.

**Table 1:** Predictions derived from the constraint hypothesis and the mating opportunity hypothesis for the co‐occurrence of female‐biased SSD and protandry in the crab spider *Mecaphesa celer*. While the mating opportunity hypothesis states that large female size/small male size are consequences of selection for protandry, the constraint hypothesis states that protandry is a side effect of selection for small male size/large female size

Predictions	Mating opportunity hypothesis	Constraint hypothesis
(1) Maturation time	Males mature synchronously and prior to females	Males tend to mature prior to females, low synchrony
(2) Female mating status	Proportion of virgin females decreases rapidly along the season	Proportion of virgin females is unrelated to timing along the season
(3) Female reproductive success	Reproductive success related to timing of maturation: early‐matured females more fecund than late matured females	Reproductive success related to female size: large females more fecund
(4) Size versus Timing of maturation	Size directly related to timing of maturation: Early‐matured individuals are smaller than late matured individuals	

## 2 METHODS

### 2.1 Field observations

We followed a population of *Mecaphesa celer* from a 20,000 m^2^ tall grass prairie patch at Holmes Lake park, Lincoln, NE, USA in 20 surveys distributed twice a week between May 13th and July 31th, 2015. Female and male *M. celer* are typically found on top of flowers during the warmest hours of the day. During each field survey, we sampled all plants bearing flowers with beat sheets and sweeping nets, starting at 12:30 h. We aimed to collect as many spiders as we could get within 4 h of collecting effort. For all *M. celer* individuals found, we recorded approximate instar (based on predetermined size categories), developmental status (mature/not mature, determined by the opening of the females’ epigynum and the pigmentation of the males’ pedipalp bulb), and sex (female, male, or unknown. Female and male spiders are easily distinguishable, but sexual dimorphism becomes apparent only after the fifth instar. Individuals younger than that were therefore classified as “unknown”). To obtain accurate measurements of size, we placed each individual in flat 2 × 2 cm sealable plastic bags and photographed them against millimeter graph paper. At the end of each survey trip, we released all spiders in the general area and on the flower type of their original collection.

To estimate the likelihood of encountering a virgin female along the season, on the last survey of each week, we randomly selected five to eight adult females to bring to the laboratory. These females were maintained in the laboratory and observed for the production of an egg sac—an indication of being previously mated. We calculated the proportion of females collected each week that laid fertilized egg sacs in the laboratory and used it as a proxy for the proportion of females that were already mated in the field that week. In the laboratory, we housed these females individually in 4 × 4 × 6 cm acrylic cages in a room at 26°C and 60% relative humidity, under a 14:10 light: dark cycle. We provided them with ad libitum water and small pieces of netting for perching. We fed field‐collected females twice a week with two juvenile crickets (*Acheta domesticus*, 1 mm; Ghann's Cricket Farms, GA, USA), and monitored them every 2 days to check for egg sacs. Once females laid their egg sacs, we stopped feeding them until the spiderlings had hatched and dispersed (females guarding egg sacs will not eat, and crickets may prey upon eggs—M.‐C. Chelini, pers. obs.). Upon spiderling dispersion (3–5 days after egg sac hatching), we separated them from the mother, counted them, and sacrificed them by freezing. We returned the mothers to their cages and to their normal feeding schedule until they laid another egg sac, or until their natural death. We sacrificed all remaining females by freezing on the 18th of December 2015, after temperatures in the field had dropped below freezing.

To determine the relationship between the timing of male maturation and degree of SSD, we measured all adult individuals found during each field survey. We measured each female's and male's cephalothorax width (the most appropriate measure of body size in spiders with SSD—Foellmer & Moya‐Laraño, 2007) on the photographs taken in the field using the software Image J (Rasband 1997–2012).

### 2.2 Statistical analyses

#### 2.2.1 Prediction 1—Timing of male maturation

We tested whether males mature earlier than females in the field with a binomial generalized linear model (GLM), using the proportion of adult individuals as a response variable and the individuals’ sex, the Julian date of each survey, and their interaction as predictor variables.

#### 2.2.2 Prediction 2—Females mating status

We tested whether the proportion of virgin females decreases along the reproductive season with a binomial GLM, with the proportion of females brought to the laboratory that did not lay an egg sac (i.e., were likely still virgin, as egg sac laying is the best indicator of a female's mating status) as the response variable and the Julian date as the predictor variable. To combine graphically these laboratory results with our field observations, we multiplied the proportion of gravid females in the laboratory by the number of females found in the field, obtaining a rough estimation of how many females could already be mated at any given time in the field.

#### 2.2.3 Prediction 3—Females reproductive success

Males could benefit from mating with early‐maturing females if these are more fecund than late‐maturing females (Carvalho, Queiroz, & Ruszczyk, 1998), or simply due to the fact that early‐maturing females may have more time along the season to invest in multiple egg sacs (Aebischer et al., 1996). Although *M. celer* females may lay multiple egg sacs in the laboratory (Chelini & Hebets, 2016a, 2016b; Muniappan & Chada, 1970), we do not know how likely this occurrence is in nature. We tested whether timing along the season influences the number of spiderlings each female had using a linear model (LM) with (1) the number of spiderlings hatching from their first egg sac or (2) the total number of spiderlings each female had (adding up multiple egg sacs) as the response variables and the Julian date in which each mature female was collected (proxy for female maturation date) as the predictor variable.

We also tested the relationship between female size and spiderling number. Previous studies have demonstrated that number of spiderlings and number of eggs are very highly correlated in *M. celer*, and fertilization success (i.e., the proportion of eggs that hatches successfully) averages 97% (Chelini & Hebets, 2016a, 2016b). Number of spiderlings is therefore a valid proxy of fecundity in *M. celer* (Chelini & Hebets, 2016b). For this second analysis, we used two LMs with (1) number of spiderlings hatched from the first egg sac or (2) the total number of spiderlings each female had (adding up multiple egg sacs) as the response variable and each female's cephalothorax width as the predictor variable. We analyzed Predictions 1, 2, and 3 with the functions *glm* from R's package *lme4* (Bates, Maechler, Bolker, & Walker, 2015; R Development Core Team, 2014).

#### 2.2.4 Prediction 4—Timing of maturation and size at maturity

If SSD is related to protandry, we expect early maturation to be related to smaller male size. As such, we predicted that early‐maturing males are smaller than late‐maturing males. We tested the relationship between size and timing of maturation in *M. celer* with two generalized additive models (GAMs), one for females and one for males, using the adult individuals’ cephalothorax width as a response variable and a smooth function of the Julian date as a predictor variable. These analyses were conducted with the function *gam* from R's package *mgcv* (R Development Core Team, 2009; Wood, 2011).

### 2.3 Calculating female and male probability of reproductive success along the season

The probability of encounter between two random individuals depends on the population's relative density (Kokko & Rankin, 2006). In our dataset, population density was at its highest when we collected the highest number of spiders in a 4 hr time period (*N*_max_ = 96). For calculation purposes, we considered that when the density of the population is at its highest (96 individuals), the relative density of the population is one. The relative density of the population throughout the season (*N*_rel_) is therefore calculated as

(1) $$N_{\mathrm{rel}}=\frac{N_{t}}{N_{\max}},$$

where *N*_*t*_ is the number of spiders collected on that date, for all Julian dates *t*.

Assuming that a given female in the population is virgin, the probability that she will succeed in being found by a male is calculated as a function of the relative density of the population, *N*_rel_ and the probability of finding a mature male in the field at that time, *P*_male_. Male spiders require a time interval close to 24 h between copulations in order to recharge their pedipalps (spiders’ copulatory organs) (Morse, 2007). As such, we include the probability that this male has not found a female on the same date, *1−P*_fem_, in our function of female success. The probability of success for females, PS_fem_, is therefore calculated as

(2) $${\text{PS}}_{\mathrm{fem}}=N_{\mathrm{rel}}\times P_{\mathrm{male}}\times \left(1-P_{\mathrm{fem}}\right).$$

The probability that a male will succeed in finding a receptive mate is a function of the relative density of the population, *N*_rel_, the probability of finding a mature female, *P*_fem_, the probability that this female was virgin on that date, *P*_virgin_, and the probability that this female has not been found by another male before, 1−*P*_male_, as *M. celer* females seldom remate (Chelini & Hebets, 2016a, 2016b). In contrast to females, male *M. celer* may mate multiply (Chelini & Hebets, 2016a) and males that mature earlier in the season may potentially mate with a higher number of females than late‐maturing males. In order to account for this difference in potential opportunities for reproduction along the season, we added a weighting factor (*w*(*t*)) to our function of male success. For simplicity, we chose this weighting factor to be a function of time that is maximal and equal to one early in the season, and decreases linearly to near zero at the very end of the season:

(3) $$w\left(t\right)=\left\{\begin{array}{c} 1,t<140\\ 0.01+0.99\times \frac{t_{f}}{t_{f}-t_{i}}-t\times \frac{0.99}{t_{f}-t_{i}},t\geq 140 \end{array}\right.,$$

with *t*_f_ being the last day of the season and *t*_*i*_ being the Julian date when the first mature male was found (i.e., the starting point of the males’ season—see Fig. S1). The probability of success for males, PS_male_, is, therefore:

(4) $${\text{PS}}_{\mathrm{male}}=N_{\mathrm{rel}}\times P_{\mathrm{fem}}\times P_{\mathrm{virgin}}\times \left(1-P_{\mathrm{male}}\right)\times w\left(t\right).$$

## 3 RESULTS

### 3.1 Prediction 1—Timing of male maturation

**Figure 1:**
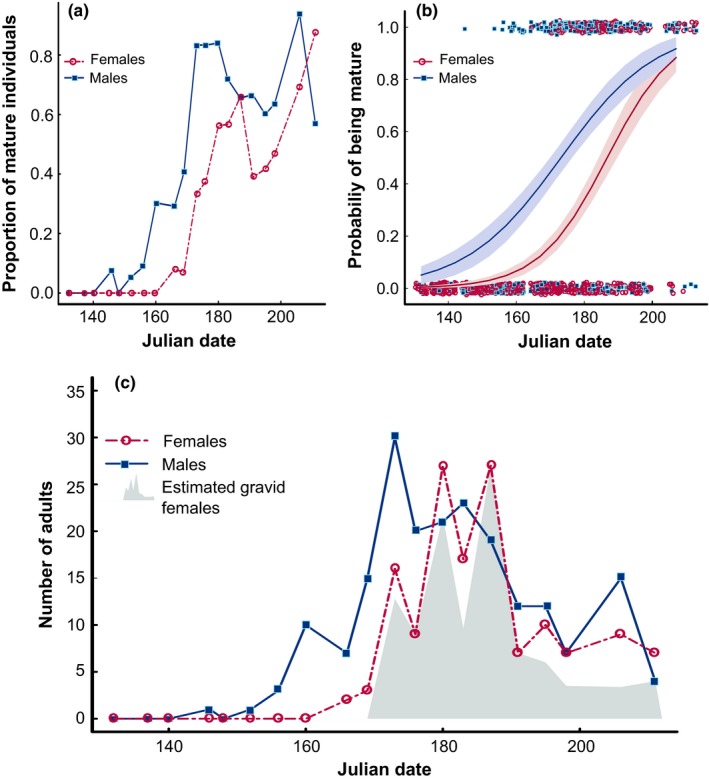
(a) Proportion of collected *Mecaphesa celer* individuals of each sex that was mature in each week of the reproductive season (May 13th to July 31st, 2015); (b) Probability that a sampled female and male *Mecaphesa celer* individual was sexually mature in the wild during the reproductive season. Lines indicate the probability slope predicted by a binomial GLM and the shaded areas correspond to the 95% confidence intervals; (c) Number of adult females and males per sex along the season. The gray area corresponds to the estimated number of mated females, based on the proportion of females collected in each week that laid a fertilized egg sac in the laboratory

We sampled a total of 1,340 juvenile and adult *M. celer* throughout the season. In each field survey, we collected between 37 and 96 individuals, with numbers declining abruptly from mid to late July (late season). Male *M. celer* mature significantly earlier than females in the wild, but with only moderate degrees of synchrony. The proportion of mature males changed from 0% to 85% in approximately 25 days (Table 2, Figure 1). The operational sex ratio was male‐biased throughout most of the season, with the exception of 2 weeks in which females were the most abundant sex (Figure 1c).

**Table 2:** Binomial GLM model on the probability of being mature according to sex and time along the season

	Estimate	*SE*	*z*	*p*
(Intercept)	−18.70	1.58	−11.82	<2.00E‐16
Sex	6.35	2.12	2.99	.002
Julian date	0.10	0.01	11.35	<2.00E‐16
Sex × Julian date	−0.03	0.01	−2.40	.02

Residual deviance = 1,355.31, *df* = 3, Deviance = −449.81, *p *<* *2.2e‐16.

### 3.2 Prediction 2—Females mating status

**Figure 2:**
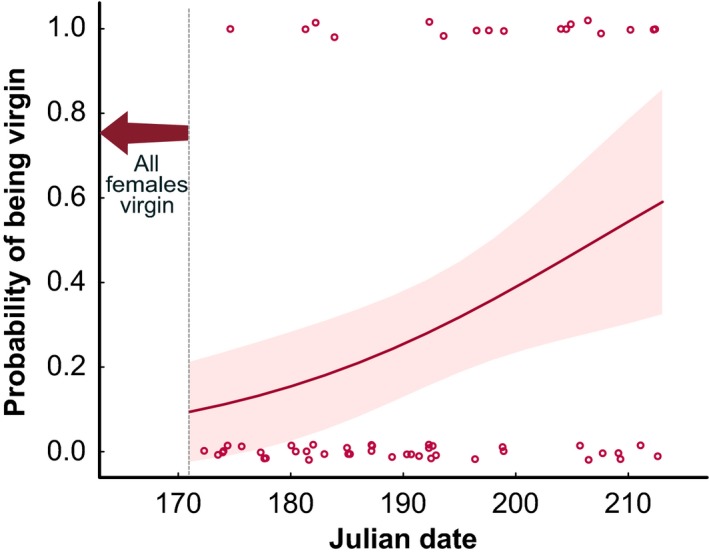
Probability of mature females being virgin along the reproductive season. All females are virgin at the start of the season but become very rapidly mated. The solid line represents the probability as predicted by the binomial GLM, and the shaded red area represents the 95% confidence interval

All *M. celer* females start the season as virgins, given that this is a univoltine species that lives for only 1 year (Muniappan & Chada, 1970). Female maturation was less synchronous than male maturation. The proportion of mature females increased from 0% to 65% in approximately 25 days. The proportion of virgin females (i.e., females that did not lay egg sacs in the laboratory) became immediately low early in the season, indicating that females are rapidly mated, but then increased significantly until late July (Table 3, Figure 2).

**Table 3:** Binomial GLM model on the proportion of mated females along the season. The proportion of mated females decreases with time

	Estimate	*SE*	*z*	*p*
(Intercept)	12.9669	5.13515	2.525	.011
Julian date	−0.0626	0.02627	−2.383	.017

Residual deviance = 69.46, *df* = 1, Deviance = 6.38, *p* < .02.

### 3.3 Prediction 3—Females reproductive success

*Mecaphesa celer* females collected in the field that laid eggs in the laboratory had an average of 112 spiderlings (min = 14, max = 209). Of those, an average of 86.2 hatched from the first egg sac (min = 29, max = 168). Clutch success (number of spiderlings/total number of eggs) in female *M. celer* averages 97% (Chelini & Hebets, 2016b), so this variance in spiderling numbers is not due to differences in fertilization success, but rather to differences in total number of eggs. The date of each female's collection (proxy for her maturation date) was not related to the number of spiderlings hatching from her first egg sac (LM: *F* = 0.056, *df* = 38, Residual st. error = 33.64, Multiple *R* ^2^ = 0.001, *p* = .82), or to her total number of spiderlings (LM: *F* = 0.038, *df* = 39, Residual st. error = 50.2, Multiple *R* ^2^ = 0.001, *p* = .85).

Female size was marginally related to the number of spiderlings hatched from the first egg sac (LM—Table 4), but not to the total number of spiderlings hatching from multiple egg sacs (LM: *F* = 1.94, *df* = 34, Residual st. error = 49.34, Multiple *R* ^2^ = 0.054, *p* = .17).

**Table 4:** Linear model (LM) on the relationship between *M. celer* females’ size (cephalothorax width) and total number of spiderlings (LM:* F *=* *3.853, *df* = 33, Residual st. error = 33.58, Multiple *R* ^2^ = 0.11, *p *=* *.058

	Estimate	*SE*	*z*	*p*
Intercept	−8.70	49.06	−0.177	.86
Number of spiderlings	40.24	20.50	1.963	.058

### 3.4 Prediction 4—Timing of maturation and size at maturity

**Figure 3:**
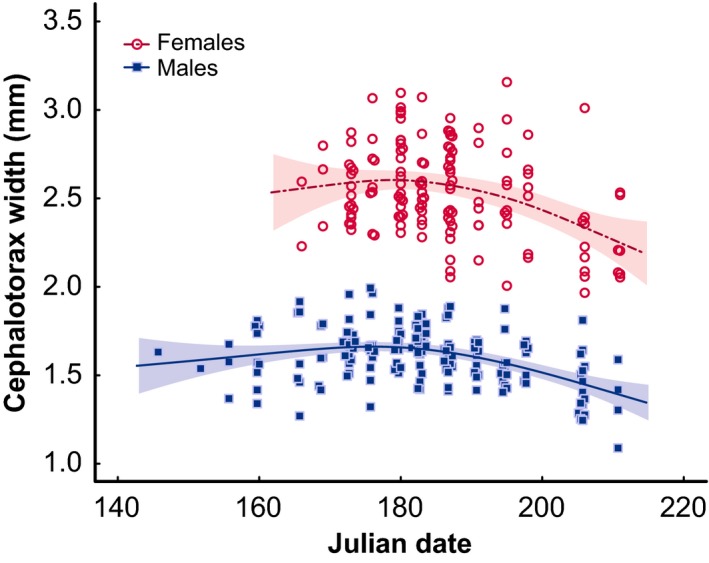
Female and male *Mecaphesa celer* size throughout the season. Lines represent the values predicted by a GAM. Red and blue shaded areas represent female and male 95% confidence intervals

Across the 338 adult female and male *M. celer* individuals that we measured throughout the season, female and male size peaked early in the season, in mid to late June, then decreased from late June to late August (Males GAM: *F* = 14.76, *p* = 3.06e‐09, deviance explained = 21.7%; Females GAM: *F* = 8.25, *p* = 7.07e‐05, deviance explained = 15.4%). The degree of SSD (average female/average male size ratio) varied from 1.48 in mid‐June to 1.66 in late August (Figure 3).

### 3.5 Female and male probability of reproductive success along the season

**Figure 4:**
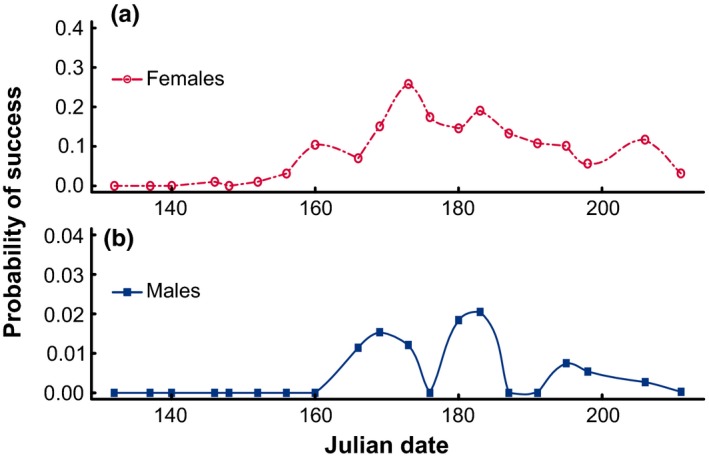
(a) Probability of a female *M. celer*'s success at being found by a male along the season; (b) Probability of a male *M. celer*'s success at finding a virgin female along the season

Our model indicates that a female's probability of reproductive success closely follows the proportion of adult males in the population along the season (Figure 4a). A male's probability of reproductive success, however, is less straightforward and seems to peak in three different moments: (1) early in the season, (2) mid‐season, and (3) a smaller peak in late season (Figure 4b). Note that comparing the magnitude of these probabilities is only valid within sexes, and not between sexes, as the parameters defining these probabilities take into account sex‐specific mate search peculiarities, such as likelihood of encountering virgin females (see Section 2), and as such are not the same for females and males.

## 4 DISCUSSION

Field data collected throughout the reproductive season on a population of the female‐biased SSD crab spiders *Mecaphesa celer* demonstrate that this species is indeed protandrous—males mature on average significantly earlier than females. Early‐maturing females mate quickly, as the majority of our early field‐collected females produced fertilized egg sacs. Surprisingly, the proportion of gravid females decreased throughout the season. Timing of female maturation was not correlated with offspring numbers, but female size tended to influence positively the first clutch size. Additionally, size measurements show that early maturation does not dictate small male size, as early‐maturing individuals of both sexes were significantly larger than late‐maturing ones. Pooling our results into a model predicting female and male reproductive success throughout the season indicated no clear benefit of early male maturation, but rather the existence of at least two male strategies with comparable benefits. Together, our results provide mixed support for both hypotheses linking protandry and the evolution of SSD in *M. celer*, and suggest that more than one source of selection may be at play.

Under a scenario of adaptive protandry (i.e., mating opportunity hypothesis), we predicted a high degree of synchrony in both male and female maturation times, and strong competition among males for access to virgin females early in the season. Male maturation is not strongly synchronous, as the first peak in male maturation is spread over a couple of weeks. Female maturation was also not highly synchronous, but rather distributed over a couple of weeks early in the season, and in a second peak at the very end of the season. Moreover, while early‐maturing females became very rapidly mated (i.e., produced a viable egg sac when brought back to the laboratory), over half of the late‐maturing females remained unmated. Late‐maturing males could therefore benefit from lower degrees of competition for access to virgin mates (Kasumovic & Andrade, 2009).

Refuting our third prediction of the mating opportunity hypothesis, we found no evidence that maturing early in the season correlates with higher lifetime fecundity. Larger females, however, tended to produce more offspring than smaller females, supporting the predictions of the constraint hypothesis. Both sexes’ size peaked toward the middle of the season, with late‐maturing individuals being significantly smaller than early‐ to mid‐maturing individuals (refuting our fourth prediction for both hypotheses). In arthropod species with winter diapause, such as *M. celer*, late‐born individuals are likely constrained to shorten and/or speed up their development to mature prior to the end of the reproductive season, maturing at a smaller size than early‐born ones (Abrams et al., 1996; Goulson, 1993; Johansson & Rowe, 1999). Our results align with these predictions, indicating that both sexes regulate the timing of their development based upon the progression of the season (Gunnarsson & Johnsson, 1990; Morbey, 2013). Moreover, the difference in maturation times between females and males is much shorter in the field that what we observe in laboratory conditions (Chelini, DeLong and Hebets et al., in prep.). Female and male growth rates are therefore not invariable, but rather highly plastic, being influenced by environmental factors. The constraint hypothesis depends on a positive relationship between body size and maturation date (i.e., size increasing along with timing of maturation, Morbey, 2013). In species where timing of maturation and body size are either unrelated or where early‐matured males are larger, such as *M. celer*, it is highly unlikely that protandry evolved simply as an incidental by‐product of selection for SSD, refuting the constraint hypothesis (Cueva del Castillo & Nunez‐Farfan, 1999; Nylin, Wiklund, Wickman, & Garcia‐Barros, 1993; Wong‐Nunoz, Cordoba‐Aguilar, Cueva del Castillo, Serrano‐Meneses, & Payne, 2011; Zonneveld, 1996).

Interestingly, the plasticity of female and male growth trajectories, evidenced by females and males ability to regulate their timing of development, and consequently adult size, based on the progression of the season, also calls into question the relationship between the mating opportunity hypothesis and SSD: If females and males can adjust their growth rate and therefore adult size based on environmental variables, selection for early male maturation would not necessarily lead to such extreme degrees of female‐biased SSD as seen in spiders. In other words, the difference in adult female and male size (often more than twofold in magnitude) is not consistent with selection for males to mature a mere few days prior to females (see theoretical predictions of Nylin et al., 1993). If organisms are able to make adaptive decisions about their growth rate (Abrams et al., 1996), protandry and SSD may evolve independently, and protandry may co‐occur with any degree and direction of SSD (e.g., female‐biased, male‐biased or neutral) (Berner & Blanckenhorn, 2006; Morbey, 2013). Female‐biased SSD is therefore also unlikely to be simply an epiphenomenon of selection for the increased mating opportunities provided by protandry. The question of how adaptive is protandry, i.e., if early male maturation does indeed increase mating opportunities, remains nonetheless crucial.

For late‐born males, the most adaptive strategy seems to be to shorten their development time, maturing at a smaller body size, in order to have access to late‐maturing females. Maturing late in the season is not, however, without its costs. The most evident cost relates to changes in the population density. Population density is well known to have a profound impact on individual reproductive success and on mating systems as a whole (Kokko & Rankin, 2006). The number of females in a population that die unmated, as seems to be the case for many late‐maturing *M. celer* females, is expected to increase as the population density decreases (Calabrese et al., 2008; Morse, 2013). For female crab spiders, that do not spin pheromone‐loaded webs, or any animal not known to release sex‐specific pheromones (Anderson & Morse, 2001; Dodson & Schwaab, 2001; Leonard & Morse, 2006; Morse, 2010), low population densities make mate search particularly challenging. As such, *M. celer* males likely face a trade‐off between high male–male competition early in the season (as evidenced by the high proportion of females that become mated immediately after maturation) and costly mate search toward the end of the season (as evidenced by the low population densities).

Early male maturation is likely associated with benefits other than simply higher female density. First, early‐maturing males may have access to larger and potentially more fecund females (Blanckenhorn, 2000; Honek, 1993; Nali, Zamudio, Haddad, & Prado, 2014; Preziosi, Fairbairn, Roff, & Brennan, 1996). Offspring from early‐maturing males are also likely to hatch sooner and have a longer period of time to forage before entering winter diapause (Cherrill, 2002; Landa, 1992), achieving larger sizes, greater survival, and reproductive success than the offspring of late‐maturing males (e.g., Einum, Fleming, & Inum, 2000; Varpe, Jørgensen, Tarling, & Fiksen, 2007). Finally, male lifespan also influences the benefits obtained through protandry, as it determines the length of the males’ reproductive season (Morbey & Abrams, 2004; Morbey & Ydenberg, 2001; Wiklund & Fagerström, 1977). Sexual cannibalism is relatively infrequent in *M. celer*, males may mate multiply (Chelini & Hebets, 2016a) and, in laboratory conditions, males can live for more than 2 months (M.‐C. Chelini, pers. obs.). Early maturation thus may grant males access to a larger number of virgin females throughout the entire season, and not simply during the first peak in female maturation (Canal, Jovani, & Potti, 2012; Wiklund & Fagerström, 1977). In sum, *M. celer* may males have multiple avenues through which they benefit by maturing early in the season, despite facing higher male–male competition.

Our modeling of the probabilities of male reproductive success sheds light on the balance between male–male competition, female density, and number of reproductive opportunities in *M. celer*. According to our model, *M. celer* males can optimize their reproductive success through more than one strategy. Males maturing toward the middle of the season, past the first burst of female maturation, benefit from much lower degrees of male–male competition and have comparable, if not higher, chances of success than early‐maturing males. Late‐maturing males have a reduced probability of success when compared to early‐ and mid‐maturing males. In many taxa, competitively superior large males mature sexually or arrive at the breeding grounds at the time where chances of finding a mate are at their highest, that is, prior to smaller and competitively inferior males (Kokko, 1999; Maklakov et al., 2004; but see Alcock, 1997; Candolin & Voigt, 2003 for the reverse pattern). Our results fit this pattern, with the largest males maturing mid‐season. *Mecaphesa celer* males could potentially be following conditional strategies with unequal fitness benefits, where large males follow the highest rewards strategy, and smaller, late‐born males do “the best of a bad lot” (Candolin & Voigt, 2003; Eberhard, 1982; Morbey, 2013). In *M. celer*, reaching sexual maturation toward the middle of the season and at a large body size could represent an optimal strategy, contradicting the mating opportunity hypothesis for the joint evolution of protandry and SSD.

Our results with *M. celer* indicate that protandry is not an incidental by‐product of selection for large females size (Vollrath & Parker, 1992; Elgar & Bathgate, 1996; Legrand & Morse, 2000; Kasumovic & Andrade, 2009; Danielson‐François et al., 2012; Neumann & Schneider, 2015; but see Maklakov et al., 2004). Adaptive protandry, in turn, does not seem to be the single driver of female‐biased SSD in spiders. We draw attention to the fact that flexible growth rates, as those of many spiders, dissolve the evolutionary link between protandry and female‐biased SSD. The hypothesized relationship between SSD and degree of protandry has been theoretically (Nylin et al., 1993) and empirically shown to be weak in a variety of arthropods species, including spiders (Berner & Blanckenhorn, 2007; Blanckenhorn et al., 2007; Cueva del Castillo & Nunez‐Farfan, 1999; Gunnarsson & Johnsson, 1990; Maklakov et al., 2004). Small male size may, therefore, be directly selected for either through differences in survival (De Mas, Ribera, & Moya‐Laraño, 2009; Vollrath & Parker, 1992), increased agility (Corcobado, Rodríguez‐Gironés, De Mas, & Moya‐Laraño, 2010), or simply smaller foraging requirements (Blanckenhorn, Preziosi, & Fairbairn, 1995; Yasuda & Dixon, 2002). We urge future studies on the evolution of female‐biased SSD to go beyond the hypothesized relationship between size and timing of maturation, by understanding the evolutionary drivers of large female size, small male size, and their combination (Chelini & Hebets, 2016a).

## CONFLICT OF INTEREST

None declared.

## AUTHOR'S CONTRIBUTIONS

M.‐C. Chelini and E. Hebets conceived the ideas and designed methodology; M.‐C. Chelini collected and analyzed the data and led the writing of the manuscript; E. Hebets contributed critically to the drafts and gave final approval for publication.

## DATA ACCESSIBILITY

Upon acceptance of this manuscript, data will be made available in Dryad Digital Repository.
