# Supplementary material for: Field evidence challenges the often‐presumed relationship between early male maturation and female‐biased sexual size dimorphism
Source: Ecol Evol. 2017 Oct 16;7(22):9592–601. doi: 10.1002/ece3.3450 (PMC5696407; doi:10.1002/ece3.3450)
Supplement: Supplementary file 1 [file ECE3-7-9592-s001.docx]

Fig. S1: Weighing function (*w*(t)) incorporated in the calculations of male probability of mating success along the season. To take into account the effect of male longevity in the number of mating opportunities males maturing at different points of the season have, *w*(t) is a linear function that is maximized early in the season and decreases to near zero as the end of the season:

$w\left( t \right)= \left\{ \begin{matrix} 1, t<140 \\ 0.01+0.99*\frac{t_{f}}{t_{f}-t_{i}}-t*\frac{0.99}{t_{f}-t_{i}}, t \geq140 \end{matrix} \right.$ (eqn 3),

with t*_f_* being the last day of the season and t*_i_* being the Julian date when the first mature male was found (*i.e.*, the starting point of the males’ season).
